# Determination of the threshold of cardiac troponin I associated with an adverse postoperative outcome after cardiac surgery: a comparative study between coronary artery bypass graft, valve surgery, and combined cardiac surgery

**DOI:** 10.1186/cc6126

**Published:** 2007-09-21

**Authors:** Jean-Luc Fellahi, François Hedoire, Yannick Le Manach, Emmanuel Monier, Louis Guillou, Bruno Riou

**Affiliations:** 1 Centre Hospitalier Privé Saint-Martin, 18 rue des Roquemonts, 14050 Caen Cedex 4, France; 2 Department of Anesthesiology and Critical Care, CHU Pitié-Salpêtrière, 47-83 boulevard de l'Hôpital, 75013 Paris, France; 3 Emergency Medical Department, CHU Pitié-Salpêtrière, 47-83 boulevard de l'Hôpital, 75013 Paris, France

## Abstract

**Introduction:**

The objective of the present study was to compare postoperative cardiac troponin I (cTnI) release and the thresholds of cTnI that predict adverse outcome after elective coronary artery bypass graft (CABG), after valve surgery, and after combined cardiac surgery.

**Methods:**

Six hundred and seventy-five adult patients undergoing conventional cardiac surgery with cardiopulmonary bypass were retrospectively analyzed. Patients in the CABG (*n *= 225) and valve surgery groups (*n *= 225) were selected after matching (age, sex) with those in the combined surgery group (*n *= 225). cTnI was measured preoperatively and 24 hours after the end of surgery. The main endpoint was a severe postoperative cardiac event (sustained ventricular arrhythmias requiring treatment, need for inotropic support or intraaortic balloon pump for at least 24 hours, postoperative myocardial infarction) and/or death. Data are presented as the median and the odds ratio (95% confidence interval).

**Results:**

Postoperative cTnI levels were significantly different among the three groups (combined surgery, 11.0 (9.5–13.1) ng/ml versus CABG, 5.2 (4.7–5.7) ng/ml and valve surgery, 7.8 (7.6–8.0) ng/ml; *P *< 0.05). The thresholds of cTnI predicting severe cardiac event and/or death were also significantly different among the three groups (combined surgery, 11.8 (11.5–14.8) ng/ml versus CABG, 7.8 (6.7–8.8) ng/ml and valve surgery, 9.3 (8.0–14.0) ng/ml; *P *< 0.05). An elevated cTnI above the threshold in each group was significantly associated with a severe cardiac event and/or death (odds ratio, 4.33 (2.82–6.64)).

**Conclusion:**

The magnitude of postoperative cTnI release is related to the type of cardiac surgical procedure. Different thresholds of cTnI must be considered according to the procedure type to predict early an adverse postoperative outcome.

## Introduction

Cardiac troponin I (cTnI) is a highly sensitive and specific biological marker of myocardial necrosis [1]. In noncardiac surgery the definition of abnormal cTnI release during the postoperative period has been modified in past years, as a result of better understanding of the pathophysiological mechanisms involved in postoperative myocardial necrosis [2-4]. The most recent definition stipulates that any increase in cTnI above the normal range should be considered an indication of myocardial necrosis [5].

The problem is far more complex in cardiac surgery with cardiopulmonary bypass (CPB) since cardiac surgery *per se *induces an increase in postoperative cTnI, even in the absence of postoperative cardiac complications [6-8]. CPB is associated with a certain degree of myocardial damage, and its duration is likely to influence the postoperative cTnI release [9]. This increase could also depend on the type of surgery and its subsequent degree of direct surgical trauma [9,10]. Indeed, valve replacement may induce more direct surgical trauma than coronary artery bypass graft (CABG) surgery, whereas combined surgery is associated with a more prolonged CPB time.

Whatever the various mechanisms that explain the postoperative cTnI release in cardiac surgery, it has been shown that cTnI is an independent predictor of short-term and long-term adverse outcome in cardiac surgical patients [11,12]. The definition of the threshold of postoperative cTnI release associated with a poor outcome may therefore be of paramount importance in cardiac surgery to identify a transition in postoperative risk. At the present time there is little information concerning the methodology used to determine the threshold of postoperative cTnI, and the precision of this threshold determination has never been studied. Moreover, whether the potential differences in cTnI release among procedure types may influence, first, the threshold of cTnI associated with an adverse postoperative outcome and, second, the accuracy of cTnI to predict such an adverse outcome remains unknown. Finally, patients undergoing combined cardiac surgery are well known to have a higher risk of postoperative morbidity and mortality than those undergoing single procedures [11,13,14]. Whether this latter issue influences the accuracy of cTnI to predict a poor outcome remains also unknown.

We therefore decided to conduct a comparative study between CABG, valve surgery, and combined surgery in order to determine the postoperative cTnI release and the thresholds of cTnI that predict adverse outcome – the hypothesis tested being that both cTnI release and the thresholds would differ among procedure types. In addition, we proposed a method to evaluate the precision of the threshold determination.

## Materials and methods

### Patient population

We used a comprehensive, prospectively recorded database describing the clinical and surgical characteristics of 2,875 patients undergoing cardiac surgery with CPB at the Centre Hospitalier Privé Saint-Martin (Caen, France) from January 1999 to October 2004. An anesthesiologist (JLF) entered the data, and a systematic audit by a trained research technician who participated in previous studies [12,13] allowed verification of the accuracy in coding data. Missing data were coded as absent. The study was approved by an institutional review board (Comité Consultatif pour la Protection des Personnes se prêtant à la Recherche Biomédicale Pitié-Salpêtrière, Paris, France). Because data were collected during routine care of patients that conformed to standard procedures currently used in our institution, authorization was granted to waive written informed consent.

**Figure 1:**
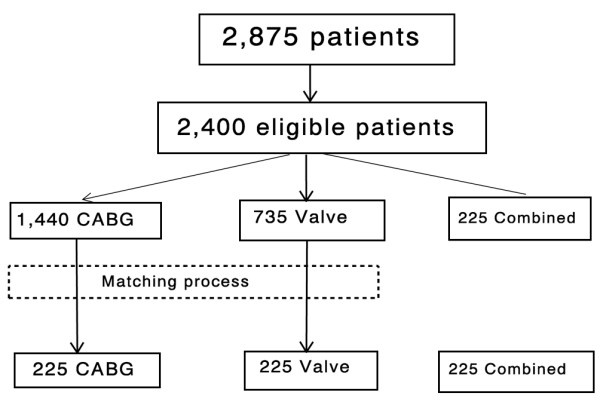
Profile of the study group. CABG, coronary artery bypass graft surgery.

Inclusion criteria were elective CABG, aortic valve or mitral valve replacement surgery, and combined surgery (CABG plus aortic valve or mitral valve replacement). Patients with increased risk of postoperative cardiac morbidity and mortality or of cTnI release (*n *= 475 patients, 17%) were excluded: emergency surgery in <24 hours (*n *= 86 patients, 3%), reoperative procedures (*n *= 58 patients, 2%), recent history (<4 weeks) of acute myocardial infarction and abnormal preoperative cTnI values >0.6 ng/ml (*n *= 101 patients, 4%), and various other surgical procedures (*n *= 230 patients, 8%) including valve repair and aortic valve plus mitral valve replacement. Among the remaining 2,400 patients, because we did not have the capability to verify accurately the characteristics of every patient in the whole population, and because the power of the study was mainly related to the sample size of the smallest group, we decided to select three groups of patients of similar size by matching the participants according to age and sex. For each patient in the combined surgery group we therefore randomly selected a matched patient in the valve surgery group and a matched patient in the CABG surgery group (Figure 1).

### Perioperative management

All patients were premedicated with oral lorazepam (2.5 mg the day before surgery and 2.5 mg on the morning of surgery). β-blocking agents were given until the day of surgery in chronically treated patients. Standardized total intravenous anesthesia (target control propofol infusion, remifentanil, and pancuronium bromide) and standard monitoring techniques (five-lead electrocardiogram with computerized analysis of the ST segment and invasive arterial blood pressure) were used in all patients and complied with routine practice at our hospital [12,13]. Antifibrinolytic therapy, either tranexamic acid (15 mg/kg twice) or aprotinin (2 × 10^6 ^KIU pre-CPB, 2 × 10^6 ^KIU in prime, and 500,000 KIU/hour during surgery) was routinely administered. CPB was performed under normothermia (>34.5°C) in all types of surgery and myocardial protection was achieved by intermittent anterograde or combined (anterograde plus retrograde) warm blood cardioplegia, as previously described [12,13]. After termination of CPB, catecholamines were used when necessary, at the discretion of the attending anesthesiologist.

All patients were admitted postoperatively into the cardiac intensive care unit (ICU) for at least 48 hours. Standard postoperative care included assessment for tracheal extubation within 1–8 hours of arrival in the ICU, blood glucose control (<8 mmol/l), intravenous heparin (200 U/kg) in patients with valve disease, and aspirin (300 mg, oral or intravenous) and a low-molecular-weight heparin (nadroparin 2,850 U, anti-activated factor X, subcutaneous; Fraxiparine^®^; Sanofi-Synthelabo, Paris, France) in patients with coronary artery disease, beginning 6 hours after surgery in the absence of significant mediastinal bleeding (>50 ml/hour). β-blocking agents were continued postoperatively in chronically treated patients.

### Measurements of cardiac troponin I concentration

Blood samples were collected the day before surgery and 24 hours after the end of surgery. This single postoperative time point was chosen in accordance with previous reports showing that serum cTnI values peak at 20–24 hours after cardiac surgery [6,7,15], and with reports showing that a single 24-hour cTnI value is a significant predictor of increased postoperative ICU stay and hospital stay [16] and is an independent predictor of short-term and long-term adverse outcome in cardiac surgical patients [11,12]. A technician who was unaware of the clinical and electrocardiogram data performed the assays. cTnI was analyzed with a sensitive and highly specific immunoenzymometric assay (AxSYM Troponin-I MEIA assay; Abbott Laboratories, Rungis, France) that detects both free troponin and complex-bound troponin. The assay allows the detection of cTnI within the range of 0.3–50 ng/ml with appropriate dilutions. Values >0.6 ng/ml were considered abnormal. The within-run coefficient of variation was 6% and the between-run coefficient of variation was 11%.

### Clinical outcome

The duration of hospitalization, the length of stay in the ICU, and the inhospital mortality were recorded. As previously described [17], to enable comparison of the duration of hospitalization and the length of stay in the ICU in different groups while taking deaths into account, we calculated the number of hospital-free days and ICU-free days within 1 month after admission, all dead patients being scored 0 hospital-free days and 0 ICU-free days. To analyze the inhospital outcome, the following postoperative variables were also recorded: duration of postoperative ventilation, Simplified Acute Physiologic Score [18], reoperation rate within hospital, and cardiac and noncardiac complications. Cardiac complications included new atrial fibrillation or flutter, sustained ventricular arrhythmias requiring treatment, requirement of an inotropic agent for at least 24 hours, use of an intraaortic balloon pump in the ICU, and postoperative myocardial infarction.

Diagnostic criteria for postoperative myocardial infarction were the appearance of new Q waves of more than 0.04 s and 1 mm deep, or a reduction in R waves of more than 25% in at least two continuous leads of the same vascular territory, as previously described [12,13]. Daily 12-lead electrocardiogram recordings were assessed by two experienced physicians blinded to the clinical and biochemical information. Postoperative noncardiac complications included stroke, gastrointestinal bleeding or ischemia, sepsis, and renal dysfunction. Postoperative renal dysfunction was defined as ≥ 30% increase in the preoperative-to-maximum postoperative serum creatinine level within 7 days after surgery [19].

### End points

Severe postoperative cardiac events and death were chosen as study endpoints. A severe postoperative cardiac event was defined as one of the following: any postoperative sustained ventricular arrhythmia requiring treatment; a need for inotropic support for at least 24 hours; a need for an intraaortic balloon pump for at least 24 hours in the ICU; or postoperative myocardial infarction as defined above and previously [12,13]. Death was defined as death at any time during the hospital stay. Causes of death were recorded and classified as cardiac (heart failure, myocardial infarction, ventricular arrhythmia) or noncardiac (hemorrhage, respiratory failure, sepsis, or other causes). Because of the rare occurrence of death, the primary endpoint was a composite endpoint defined as the occurrence of severe cardiac event and/or death.

### Statistical analysis

Following a preliminary study, we made the hypothesis that the composite endpoint occurred in 15% of patients in the CABG group and in 30% of patients in the combined surgery group. Assuming an α risk of 0.05 (including the Bonferroni correction for three groups) and a β risk of 0.10, we determined that at least 210 patients should be included in each group (NQuery Advisor 3.0; Statistical Solutions Ltd, Cork, Ireland). Nevertheless, it should be pointed out that this calculation did not refer to the main objective of the study, which was to compare the cTnI thresholds among groups. Since we were not able to provide such calculation, we decided to calculate *a posteriori *the smallest difference in the cTnI threshold (versus the CABG group) that we were able to detect in our study.

Data are expressed as the mean ± standard deviation, as the median (95% confidence interval (CI)) for nonnormally distributed variables, or as the number (percentage with its 95% CI). Comparison of several means was performed using analysis of variance and the Newman-Keuls post-hoc test, using the Kruskall–Wallis test with Bonferroni correction, or using Fisher's exact method with Bonferroni correction, as appropriate. We determined the receiver operating characteristics (ROC) curve and calculated the area under the ROC curve and its 95% CI. Comparison of areas under the ROC curve was performed using a nonpaired method.

The ROC curve was used to determine the best threshold for cTnI to predict the occurrence of severe cardiac event and/or death. The best threshold was the one that minimized the distance to the ideal point (sensitivity = specificity = 1) on the ROC curve. As this method does not provide any CI of the threshold, we realized a bootstrap analysis to obtain a calculation of the best threshold and its 95% CI. Bootstrap was performed using 1,000 random samples of 75% of the population studied. To verify that the incidence of a poor outcome (which was expected to be different among groups) did not influence the troponin thresholds, we randomly selected two subgroups of patients: one in the CABG population with a high incidence of the composite endpoint (comparable with that of the global combined surgery group), and one in the combined surgery population with a low incidence of the composite endpoint (comparable with that of the global CABG group). We then calculated the thresholds of troponin using the ROC curve and their 95% CI values using the bootstrap analysis, as indicated above. Assessment of the diagnostic performance of an elevated cTnI to predict the outcome was performed by calculating the sensitivity, specificity, positive and negative predictive values, and accuracy (defined as the sum of concordant cells divided by the sum of all cells in the two-by-two table) and their 95% CI values.

We performed a multiple, forward, stepwise logistic regression to assess variables associated with the composite endpoint (severe cardiac event and/or death). We used a limited approach and included only the significant preoperative variables in the univariate analysis (*P *value of entry = 0.10), except for two variables thought to be prognostic (diabetes, age) that were systematically included in the model. The Spearman coefficient matrix correlation was used to identify significant colinearity (>0.70) between variables. The odds ratios and their 95% CI of variables selected by the logistic model were calculated. The discrimination of the model was assessed using the calculation of the area under the ROC curve (or *c*-statistics). The percentage of patients correctly classified by the logistic model was calculated using the best threshold determined by the ROC curve. Calibration of the model was assessed using the Hosmer-Lemeshow statistic (*P *> 0.05 for no significant difference between the predictive model and the observed data) [20].

*P *< 0.05 was considered significant and all *P *values were two-tailed. Statistical analyses were performed using NCSS 2001 software (Statistical Solutions Ltd) and SPSS 13.0 software (SPSS Corporation, Chicago, IL, USA). Randomization was obtained using either the Excel random function (Microsoft, Seattle, WA, USA) or that of the SPSS software.

## Results

The three groups of patients differed according to the incidence of diabetes, previous stroke, hypertension, preoperative medications taken, and durations of CPB and aortic cross-clamping (Table 1). The postoperative outcome also differed between groups, as shown by a higher incidence of severe cardiac events, and decreased values of ICU-free and hospital-free days in combined surgery (Table 2). As expected, the rare occurrence of death precluded any powerful analysis of inhospital death (Table 2).

**Table 1:** Baseline characteristics of patients undergoing coronary artery bypass graft, valve surgery, or combined cardiac surgery

Characteristic	Coronary artery bypass graft (*n *= 225)	Valve surgery (*n *= 225)	Combined surgery (*n *= 225)
Age (years)	73 ± 8	73 ± 8	73 ± 8
Men	145 (64)	145 (64)	145 (64)
Women	80 (36)	80 (36)	80 (36)
Body mass index (kg/m^2^)	27.0 ± 3.9	26.8 ± 4.0	26.4 ± 4.0
Euroscore	5 (4–5)	5 (5-5)	7 (6–7)* ^†^
Diabetes mellitus	45 (20)	24 (11)*	44 (20)^†^
Chronic obstructive pulmonary disease	21 (9)	33 (15)	13 (6)^†^
Hypertension	169 (75)	134 (60)*	145 (64)*
Stroke	19 (8)	5 (2)*	10 (4)*
Left ventricular ejection fraction (%)	65 ± 12	65 ± 10	64 ± 13
Serum creatinine (μmol/l)	100 ± 42	97 ± 19	100 ± 40
Creatinine clearance (ml/min)	61 ± 20	62 ± 20	60 ± 21
Preoperative medication			
Nitrates	166 (52)	54 (24)*	66 (29)*
Calcium blockers	76 (34)	35 (16)*	56 (25)* ^†^
β-blockers	144 (64)	44 (20)*	83 (37)* ^†^
Renin – angiotensin system inhibitors	102 (45)	93 (41)	66 (29)* ^†^
Diuretics	31 (14)	93 (41)*	114 (51)* ^†^
Surgery			
Cardiopulmonary bypass time (min)	90 ± 22	99 ± 20*	138 ± 27* ^†^
Aortic cross-clamping time (min)	46 ± 13	68 ± 13*	96 ± 19* ^†^

Data expressed as the mean ± standard deviation, the number (%), or the median (95% confidence interval). *P < 0.05 versus coronary artery bypass graft; ^†^*P *< 0.05 versus valve surgery. No statistical analysis was performed on age and sex, which were matching variables between groups.

**Table 2:** Postoperative outcome in patients undergoing coronary artery bypass graft, valve surgery, or combined cardiac surgery

Outcome	Coronary artery bypass graft (*n *= 225)	Valve surgery (*n *= 225)	Combined surgery (*n *= 225)
Duration of postoperative ventilation (hours)	7 (7-7)	7 (7-7)	7 (6–7)
Intensive care unit-free days	26 ± 4	26 ± 4	24 ± 7* ^†^
Hospital-free days	21 ± 4	21 ± 5	20 ± 6*
Simplified Acute Physiologic Score II score	30 (29–30)	30 (29–30)	30 (29–31)
Reoperation	5 (2)	5 (2)	10 (4)
Cardiac complications			
New-onset atrial fibrillation	73 (32)	86 (38)	74 (33)
Ventricular arrhythmia	14 (6)	19 (8)	37 (16)* ^†^
Inotropic support and/or intraaortic balloon pump >24 hours	24 (11)	35 (16)	44 (20)*
Myocardial infarction	8 (4)	3 (1)	22 (10)* ^†^
Noncardiac complications			
Stroke	3 (1)	4 (2)	3 (1)
Gastrointestinal	2 (1)	9 (4)	4 (2)
Sepsis	10 (4)	11 (5)	22 (10)*
Renal dysfunction	25 (11)	34 (15)	42 (19)
Severe cardiac event^a^	33 (15)	47 (21)	75 (33)* ^†^
Inhospital death	3 (1)	5 (2)	10 (4)
Severe cardiac event and/or death	33 (15)	48 (21)	77 (34)* ^†^

Data expressed as the median (95% confidence interval), the mean ± standard deviation, or the number (%). *P < 0.05 versus coronary artery bypass graft; ^†^*P *< 0.05 versus valve surgery. ^a^Defined as ventricular arrhythmia and/or inotropic support for at least 24 hours/intraaortic balloon pump for at least 24 hours and/or myocardial infarction (see Materials and methods).

Postoperative cTnI values were lacking in 29 patients (4%). The median postoperative values of cTnI were significantly different among groups (Table 3). These differences remained significant when patients with severe cardiac event and/or death were excluded (Table 3). We calculated that we had the power (80%) to detect a difference of cTnI of at least 0.6 ng/ml, compared with the CABG group. There were no significant differences among groups in the area under the ROC curve, whereas there were significant differences in the threshold of cTnI predicting either severe cardiac event and/or death (Table 4). Despite the use of a specific threshold in each group, the accuracy of cTnI was greater in the CABG surgery group than in the valve surgery or combined surgery groups (Table 5). The specificity and the negative predictive value were significantly less in the combined surgery group than in the CABG group (Table 5).

**Table 3:** Postoperative cardiac troponin I in patients undergoing coronary artery bypass graft, valve surgery, or combined cardiac surgery

	Coronary artery bypass graft	Valve surgery	Combined surgery
All patients			
*n*	215	215	216
Cardiac troponin I (ng/ml)	5.2 (4.7–5.7)	7.8 (7.6–8.0)*	11.0 (9.5–13.1)*^†^
Elevated cardiac troponin I^a^	49 (27)	77 (36)*	101 (47)*^†^
Patients without severe cardiac event or death			
*n*	183	168	142
Cardiac troponin I (ng/ml)	5.1 (4.1–5.2)	7.8 (7.3–7.8)*	9.1 (8.0–10.1)*^†^
Elevated cardiac troponin I^a^	37 (20)	48 (29)	47 (33)*
Patients with severe cardiac event or death			
*n*	32	47	74
Cardiac troponin I (ng/ml)	13.7 (6.8–30.4)	14.1 (8.1–17.8)	16.5 (14.7–19.2)*^†^
Elevated cardiac troponin I^a^	22 (69)	29 (62)	54 (73)*

Data expressed as the median (95% confidence interval) or the number (%).* *P *< 0.05 versus coronary artery bypass graft; ^†^*P *< 0.05 versus valve surgery. ^a^Above the threshold in each group as indicated in Table 4.

**Table 4:** Analysis of the receiver operating curve (ROC) and determination of the thresholds of cardiac troponin I predicting the occurrence of postoperative severe cardiac event and/or death in patients undergoing coronary artery bypass graft surgery, valve surgery, or combined cardiac surgery, and in the global population

	Area under the ROC curve	Cardiac troponin I threshold (ng/ml)
Coronary artery bypass graft (*n *= 215)	0.777 (0.683–0.871)	7.8 (6.7–8.8)
Valve surgery (*n *= 215)	0.661 (0.559–0.763)	9.3 (8.0–14.0)*
Combined surgery (*n *= 216)	0.707 (0.634–0.780)	11.8 (11.5–14.8)*^†^
Global population (*n *= 646)	0.740 (0.693–0.787)	10.4 (8.9–11.8)

Data expressed as the median (95% confidence interval). Bootstrap analysis (1,000 random sample of 75% of the population, see Materials and methods). *P < 0.05 versus coronary artery bypass graft; ^†^*P *< 0.05 versus valve surgery. No statistical comparison was performed versus the global population values.

**Table 5:** Assessment of the diagnostic performance of an elevated cardiac troponin I to predict a severe cardiac event and/or death

	Coronary artery bypass graft (*n *= 215)	Valve surgery (*n *= 215)	Combined surgery (*n *= 216)	Global population (*n *= 646)
Sensitivity	0.69 (0.51–0.82)	0.62 (0.47–0.74)	0.73 (0.62–0.82)	0.69 (0.61–0.75)
Specificity	0.80 (0.73–0.85)	0.71 (0.64–0.78)	0.67 (0.59–0.74)*	0.73 (0.69–0.77)
Positive predictive value	0.37 (0.26–0.50)	0.38 (0.28–0.49)	0.53 (0.44–0.63)*^†^	0.44 (0.38–0.51)
Negative predictive value	0.94 (0.89–0.97)	0.87 (0.80–0.92)	0.83 (0.75–0.88)*	0.88 (0.85–0.91)
Accuracy	0.79 (0.72–0.83)	0.69 (0.63–0.75)*	0.69 (0.63–0.75)*	0.72 (0.68–0.75)

Data expressed as the median (95% confidence interval). *P < 0.05 versus coronary artery bypass graft; ^†^*P *< 0.05 versus valve surgery. No statistical comparison was performed versus the global population values. The threshold in each group was that indicated in Table 4.

In the CABG subgroup of patients (*n *= 100) with a high incidence of the composite endpoint (comparable with that of the global combined surgery group, 33% versus 34%; not significant), the cTnI threshold was 7.6 (95% CI, 6.7–9.7) ng/ml and was not significantly different from that of the global CABG population. In the combined surgery subgroup of patients (*n *= 175) with a low incidence of the composite endpoint (comparable with that of the global CABG group, 15% versus 15%; not significant), the cTnI threshold was 13.8 (95% CI, 12.2–15.1) ng/ml and was not significantly different from that of the global combined surgery population. The difference in cTnI threshold between these two subgroups was significant (*P *< 0.001).

We compared patients with severe cardiac events and/or death (*n *= 158) and those patients without (*n *= 517). In the univariate analysis, there were significant differences in the incidence of chronic obstructive pulmonary disease (16% versus 8%, *P *= 0.002), in treatment using diuretics (53% versus 30%, *P *< 0.001), in left ventricular ejection fraction <50% (21% versus 8%, *P *< 0.001), in Euroscore [21] (6 (5–6) versus 5 (5–6), *P *= 0.003), in creatinine clearance (53 ± 19 ml/min versus 62 ± 21 ml/min, *P *= 0.04), in CPB duration (123 ± 34 min versus 105 ± 34 min, *P *< 0.001), in type of surgery (CABG, 15% versus 85%; valve surgery, 21% versus 79%; combined surgery, 49% versus 66%; *P *< 0.001), in cTnI (15.6 (13.3–17.6) versus 7.0 (6.7–7.4) ng/ml, *P *< 0.001), and in the proportion of patients with an elevated cTnI (69% versus 27%, *P *< 0.001), according to the thresholds defined in each group. In the logistic model, only five variables were significantly associated with severe cardiac event and/or death: an elevated cTnI, a left ventricular ejection fraction <50%, treatment by diuretics, chronic obstructive pulmonary disease, and the duration of CPB (Table 6). There was no significant correlation between these variables, except for a weak correlation between cTnI and CPB duration (*R *= 0.16, *P *< 0.001). Adding interactions between variables did not improve the logistic model. The model provided good discrimination (area under the ROC curve, 0.774 (95% CI, 0.730–0.819); 71% of patients being appropriately classified) and calibration (Hosmer-Lemeshow chi-square test = 8.58, not significant).

**Table 6:** Variables associated with postoperative severe cardiac event and/or death (*n *= 628)

Variable	Odds ratio (95% confidence interval)	*P *value
Elevated cardiac troponin I^a^	4.33 (2.82–6.64)	<0.001
Left ventricular ejection fraction <50%	2.31 (1.31–4.05)	0.004
Diuretics	1.88 (1.23–2.88)	0.004
Chronic pulmonary obstructive disease	1.95 (1.05–3.64)	0.04
Cardiopulmonary bypass time (per 1 min increase)	1.0110 (1.0043–1.0175)	0.0012

^a^Above the threshold in each group as indicated in Table 4.

## Discussion

Our study demonstrates that postoperative cTnI release in conventional adult cardiac surgery with CPB depends on the type of surgery, even in the absence of an adverse postoperative outcome; that different thresholds of cTnI according to the procedure type should be considered to predict a poor outcome; and that the accuracy of cTnI to predict a poor outcome may be different among procedure types.

Postoperative cTnI release was significantly different among the three groups of surgery. cTnI was increased in patients undergoing combined surgery when compared with CABG patients and valve surgery patients, while CABG surgery was associated with the lowest postoperative cTnI level. The differences remained significant when patients with severe cardiac event and/or death were excluded from the analysis. These results suggest that the 'basal release' of cTnI in cardiac surgery depends on the type of surgery and is increased in complex and prolonged surgical procedures. A more extensive direct surgical trauma to the myocardium and an increase in both aortic cross-clamping and CPB time could explain this higher cTnI release in valve surgery, and especially in combined surgery. Our study showed significant correlation between the duration of CPB and postoperative cTnI release. By contrast, CABG *per se *does not lead to a major release of cTnI, as previously shown [22]. Since we previously found in elective CABG surgery that the overall amount of cardiac cells injured (whatever the mechanisms of myocardial tissue insult) was reflected by postoperative cTnI release and was well correlated with the short-term and long-term clinical outcome [12], it is probable that both an increase in basal postoperative cTnI release and the worst outcome are linked in combined surgery.

The postoperative cTnI thresholds that predict the occurrence of severe cardiac events and/or death were significantly different among the three groups. The threshold was significantly higher in combined surgery and was lower in CABG surgery. Again, a probable explanation is that surgical trauma is less in CABG than that in valve surgery or combined surgery and that the duration of CPB plays a role in the postoperative release of cTnI in the absence of cardiac complications. Our study highlights the necessity to consider the type of cardiac surgery in analyzing postoperative cTnI release in clinical practice. It should also be pointed out that no previous study has focused on the comparison of thresholds determined using the ROC methodology in different population. Even the 95% CI of the best threshold was not provided in any previous study [6-13,15] although this information is of paramount importance. The use of the bootstrap technique enabled us to provide both the 95% CI of the thresholds and to compare them between different populations. This method should probably be more widely used to assess the clinical relevance of new biological markers [1].

The diagnostic performance of an elevated serum cTnI concentration to predict severe cardiac events and/or death was good, whatever the type of cardiac surgery, as previously shown [11,12]. Nevertheless, despite the determination of a particular threshold in each type of cardiac surgery, the accuracy of cTnI was significantly greater in CABG than in valve surgery or combined surgery, and the specificity of cTnI was less in combined surgery than in CABG surgery. This result is not surprising since a higher 'basal' cTnI release may have masked some small releases of cTnI due to myocardial necrosis induced by causes other than direct surgical trauma and/or CPB. In contrast, an increase in postoperative cTnI after CABG surgery is more closely related to additional ischemic myocardial damage, postoperative cardiac complications, and poor outcome. An elevated postoperative cTnI release was a strong and independent predictor of severe cardiac events and inhospital death after conventional cardiac surgery, whatever the type of surgery. A high cTnI level was associated with a fourfold increase in the risk of cardiac morbidity and mortality, according to the thresholds defined in each group. This latter result is consistent with previous results from pooled adult cardiac patients [11] and with results in elective CABG surgery [12].

Some remarks must be included to assess the limitations of our study. First, although data were entered prospectively into the database, this was a retrospective study with the usual limitations associated with such methodology.

Second, the study period was long. Even if no significant changes occurred in overall anesthetic and surgical management, we cannot preclude some minor changes that could have finally influenced the postoperative outcome of the patients.

Third, the matching process we used in the present study was not designed to match the risk stratification between groups, but rather to select three groups of same size, with the same age and gender. Other important variables could then have interacted in the etiology of cardiac injury and biased the findings. From a theoretical point of view, however, because the threshold tried to separate the studied population into two subgroups (poor outcome versus good outcome), there was no reason why this threshold was influenced by the incidence of the outcome in the population. As we showed in our subgroup analysis, the fact that the proportion of poor outcome was expected to be greater in combined surgery did not influence the value of the threshold of cTnI, thus also justifying the absence of matching according to risk stratification.

Fourth, our study provides some insights into the different mechanisms involved in 'basal' and pathological postoperative cTnI release in main types of adult cardiac surgery with CPB, but do not test appropriate strategies to improve outcome in identified high-risk patients. Futures studies should address this important issue.

Fifth, our study was performed in low-risk patients. Further studies are therefore required to determine whether our results are applicable for more high-risk patients.

Finally, the study was conducted in a single centre. The threshold values we reported must probably therefore be interpreted with cautious. Moreover, the thresholds identified were those associated with occurrence of a composite endpoint defined as severe cardiac event and/or inhospital death. As death was a rare event, the thresholds described here probably do not apply to the prediction of death, which is probably associated with higher values of cTnI, as previously described [11,12], and may not apply to the prediction on a long-term basis [12]. Further studies should also address these two important issues.

## Conclusion

The magnitude of postoperative serum cTnI release in adult cardiac surgery is related to the type of surgery, and combined surgery *per se *induces greater cTnI levels, even in the absence of postoperative severe cardiac events or death. Although our study shows that postoperative cTnI permits early and accurate identification of patients at increased risk of severe cardiac complications, it also demonstrates that different thresholds of cTnI should be considered according to the type of cardiac surgery.

## Key messages

• The magnitude of postoperative cTnI release following conventional adult cardiac surgery with CPB is related to the type of surgical procedure, even in the absence of an adverse postoperative outcome. The more the procedure is complex and prolonged, the more the cTnI release is increased, irrespective of the mechanism.

• Different thresholds of postoperative cTnI release should be considered according to the procedure type in clinical practice to predict early a poor outcome following conventional adult cardiac surgery with CPB.

• Despite the determination of a particular threshold of postoperative cTnI release in each type of cardiac surgery, the accuracy of cTnI to predict a poor outcome following conventional adult cardiac surgery with CPB may be different among procedure types, being less accurate in combined surgery and in valve surgery than in coronary surgery.

## Abbreviations

CABG = coronary artery bypass grafting; CI = confidence interval; CPB = cardiopulmonary bypass; cTnI = cardiac troponin I; ICU = cardiac intensive care unit; ROC = receiver operating characteristics.

## Competing interests

The authors declare that they have no competing interests.

## Authors' contributions

J-LF conceived of the study, participated in its design and coordination, performed acquisition, analysis, and interpretation of the data, and wrote the manuscript. FH, EM and LG made substantial contributions to acquisition of data and helped to draft the manuscript. YLM performed the statistical analysis and participated in the design of the study. BR participated in the design and coordination of the study and performed the statistical analysis. All authors read and approved the final manuscript.
